# Increased Blood Plasma Levels of Methionine-Oxidized Clusterin Correlate with a Shift from Normal to Mild Cognitive Impairment and Alzheimer’s Disease Stages

**DOI:** 10.3390/antiox15020269

**Published:** 2026-02-21

**Authors:** Amina H. Tbaba, Adam S. Smith, Jackob Moskovitz

**Affiliations:** Department of Pharmacology and Toxicology, School of Pharmacy, University of Kansas, Lawrence, KS 66045, USA

**Keywords:** clusterin, methionine oxidation, Alzheimer’s disease

## Abstract

Clusterin is a chaperon protein that is involved in many physiological processes, including binding to beta-amyloid (Aβ). Recently, we showed that in Alzheimer’s disease (AD) model mice and human postmortem brains, there are elevated levels of methionine-oxidized clusterin in the disease state versus controls. These observations prompted us to investigate the possibility that elevated methionine-oxidized levels of clusterin in human blood plasma correlate with clinical diagnosis of both mild cognitive impairment (MCI) and AD stages. To achieve this goal, we have used a combination of Elisa kits for determining the total level of clusterin and methionine-oxidized clusterin in human blood plasma, enabling the quantification of a methionine-oxidized clusterin to total clusterin ratio. This ratio was correlated with the diagnostics of three groups of patients (normal controls (NL), MCI, and AD; with *n* = 44 per group). Accordingly, it was determined that there was a significant increase in the relative methionine-oxidized clusterin level in the MCI and AD groups compared to the controls. In conclusion, it is suggested that increased levels of methionine-oxidized clusterin in human blood plasma may serve as a potential marker for MCI and AD diagnosis.

## 1. Introduction

Apolipoprotein J (clusterin) is a 70 kDa component of high-density lipoproteins in human blood plasma, comprising two disulfide-linked subunits. It is highly expressed in the brain, especially in the hippocampal region. Clusterin is a glycoprotein that plays a role in multiple physiological processes and oxidative stress-related diseases, including AD. Its extracellular form is the most common type that exhibits a major chaperon function through binding to several target proteins, including Aβ [1]. In AD, clusterin seems to have an important role in modifying the aggregation of Aβ and fostering its clearance depending on the clusterin:Aβ ratio [2]. Genetically, polymorphism variants of the clusterin gene are the third-most significant genetic risk factors for late onset of AD [3]. Oxidation of the amino acid methionine to MetO can cause a conformational change that may hamper the targeted protein function. Accordingly, oxidation of specific methionine residue/s of clusterin may interfere with its chaperon function, leading to compromised biological performances of its binding partners [4]. These observations have been further corroborated in postmortem human brains, using the same analyses, showing higher protein–methionine sulfoxide (MetO) levels in AD-brains compared to a non-AD brain [4]. Recently, we demonstrated that the levels of MetO-Clusterin were elevated in AD model mice (5xFAD) and in postmortem human brains compared to controls [4]. Furthermore, in vitro methionine oxidation of clusterin compromised its chaperon activity towards Aβ_42_ compared to native clusterin [4]. These data suggest that faulty MetO-proteins, including MetO-Clusterin, accumulate in both postmortem brains of AD model mice and of humans with AD.

## 2. Materials and Methods

Blood samples were collected from human subjects and corresponding platelet-free blood plasma samples were prepared at the University of Kansas Alzheimer’s disease Research Center (KUADRC) and KU Medical School (KUMC) using commonly practiced procedures. The blood plasma samples were shipped under ice to Dr. Moskovitz’s laboratory for further processing (a total of *n* = 132 samples; 44 samples per group: normal control (NL), mild cognitive impairment (MCI), and AD). The samples were analyzed to determine their total levels of clusterin using a commercial Elisa kit (ThermoFisher Scientific; Catalog Number EHCLU) (ThermoFisher Scientific, Waltham, MA, USA). An in-house Elisa kit was created to determine the MetO-Clusterin levels in the same samples as follows: clear 96-well plates were purchased from Pierce Biotechnology (Pierce 8-Well Polystyrene Strip Plates, Corner Notch, Cat. No. 15036) (Rockford, IL, USA). A complete set of Elisa Buffers (Invitrogen Antibody Pair Buffer Kit, Cat. No. CNB0011) (Invitrogen, Carlsbad, CA, USA) was purchased and used according to the kit’s protocol. Briefly, a set of two Elisa plates was created: one with the addition of the anti-MetO antibody (capturing antibody plate) and one without this antibody for measuring the non-specific blood plasma reactions (control plate). All the analysis steps were identical for both plates, except for the anti-Meto antibody addition. Accordingly, the capturing antibody (anti-MetO IgG: ~1 mg/mL, [5]) was diluted in coating buffer (PBS) and added to the plates at 12 µg antibody/mL. After incubation for one hour at room temperature, followed by overnight incubation (12–18 h) at 2–8 °C, the plates were washed and blocked with the kit’s blocking buffer and the Universal Blocking Reagent (1X) (BioGenex) (Fremont, CA, USA) for one hour at room temperature. Thereafter, the plates were washed again and blood plasma samples were diluted (1:100) with the Kit’s blocking buffer and added to the plates. The plates were incubated for two hours at room temperature with gentle shaking (~500 rpm). At the end of this step, followed by multiple washes, the detection antibody (Human Clusterin Biotenylated antibody; ThermoFisher Catalog number: BAM29373) was added in blocking buffer to the plates and incubated for one hour at room temperature with gentle shaking (~500 rpm). Then, following multiple washes with the wash buffer, a solution of diluted Streptavidin-HRP in blocking buffer was added to the plates that were incubated for one hour at room temperature with gentle shaking (~500 rpm). At the end of the incubation, the plates were washed with the wash buffer and TMB substrate solution was added to the plates for 30 min incubation at room temperature. Then, a stop solution was added, and the absorbance was measured at 450 nm. Analyses of results: We first calculated the net MetO-Clusterin level by subtracting the value obtained per sample in the control plate from the value obtained for the same sample in the anti-MetO antibody-covered plate. For the final calculation of the ratio of the MetO-Clusterin per total clusterin of each sample, the net MetO-Clusterin value was divided by the total Clusterin value (obtained by the ThermoFisher kit).

The specificity of the anti-MetO antibody to MetO-containing proteins was established by the Western blot analyses performed on non-oxidized and oxidized pure proteins and extracts, as previously published by us [5]. Additionally, we have shown that only sera obtained from mice that were immunized with MetO-rich protein (i.e., the antigen used to produce the anti-MetO antibody) reacted with the antigen in Western blot analyses compared to sera obtained from control mice [6]. Furthermore, the reaction of the anti-MetO antibody with MetO-Clusterin was determined through immunoprecipitation experiments using brains from postmortem human brains and AD model mice [4]. The current Elisa assay used for determining the MetO-clusterin levels has the following characteristic features: There was no interaction between the capturing anti-MetO antibody and the non-oxidized human clusterin provided by the commercial Elisa kit used in our studies (ThermoFisher kit). Compared to the performance characteristics described for measuring total clusterin by the commercial Elisa kit, the Elisa kit created by us to monitor the MetO-Clusterin levels in blood plasma is about ~100 times less sensitive in the linear range (i.e., requires 100-fold versus 10,000-fold dilution of plasma in the commercial Elisa kit). Accordingly, the detection limit was determined to be 1500 pg/mL. Besides the lower dilution used in the MetO-Clusterin assay, all the other characteristics described for the commercial Clusterin Elisa kit are the same, since the same detecting antibody was used in the MetO-Clusterin Elisa kit (anti-human Clusterin antibody).

The human blood plasma samples were obtained through the University of Kansas Alzheimer’s disease Research Center (KUADRC) and KUMedical School (KUMC), which received an IRB to obtain and distribute the samples to KU researchers, including the laboratory of Dr. Moskovitz (PI).

## 3. Results

### Levels of MetO-Clusterin in Human Blood Plasma in NL, MCI, and AD Subjects

Human blood plasma samples from 132 individuals were obtained, as described above, and the subjects’ information on their status of disease (i.e., NL.MCI, AD), age, sex, and CDR grading is described in Table 1.

**Figure 1:**
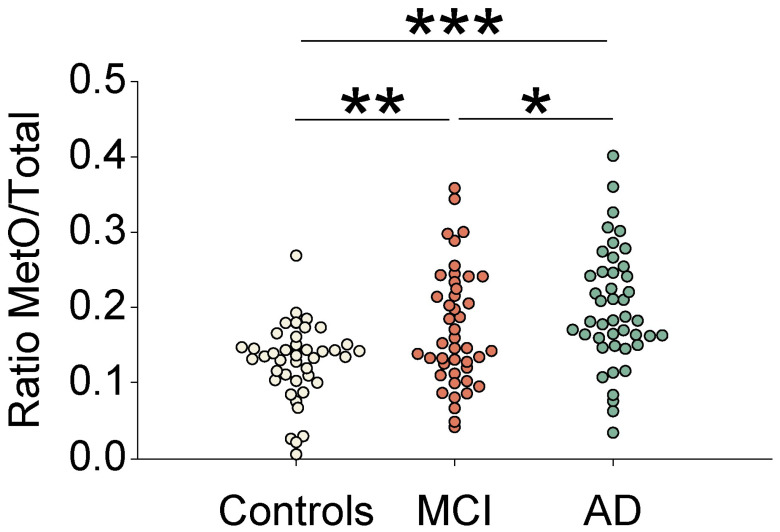
**Corelative increase in MetO-Clusterin levels in human blood plasma with MCI and AD clinical diagnoses.** Platelet-free human blood plasma samples were collected and processed from 142 individuals (44 individuals per each group: controls, MCI, and AD), as described under the “Materials and Methods” section. Diluted aliquots from each sample were used for determining the relative levels of MetO-Clusterin to total clusterin, by using Elisa assays as described under the “Materials and Methods” section. The MetO/total ratio shown in the “Y” axis was calculated by dividing the values obtained by the specific Elisa for determining the levels of MetO-Clusterin that were divided by the values obtained by the specific Elisa for the determining of the total clusterin levels. The *p* values for controls vs MCI or AD were *p* = 0.018 and *p* < 0.001, depicted with ** and *** symbols, respectively, and the *p* value for MCI vs AD was 0.05 (depicted with the * symbol). All statistical evaluations were performed by using one-way ANOVA followed by the Scheffe test. MCI: mild cognitive impairment; AD: Alzheimer’s disease.

Following the determination of the blood plasma levels of MetO-Clusterin in all the subjects according to the procedures described in the Material and Methods section, the acquired data were depicted as dependency of group of subjects (i.e., NL, MCI, and AD) (Table 1). As shown in Figure 1, in comparison to the NL group (controls), the MCI and AD showed an increase of 33% and 55% in the levels of MetO-Clu, respectively. The *p* values for NL vs MCI or AD were *p* = 0.018 and *p* < 0.001, respectively, and the *p* value for MCI vs AD was 0.05. No main effects or interactions for age, education, or race were observed in the diagnostic-significant data. There were no significant differences between the averaged values obtained for the total clusterin levels of the three subject groups. All statistical evaluations were performed by using one-way ANOVA followed by the Scheffe test. Based on Receiver Operating Characteristic (ROC) analysis, we set a cut-off value for the MetO-Clusterin ratio to 0.177 and observed an 88.5% rate (46 of 52 cases) of true positives for MCI or AD diagnosis when compared to clinical MoCA/CDR scores. This could suggest that elevated blood plasma MetO-Clusterin ratio levels are leading to an accurate clinical diagnosis of MCI or AD. However, we also found the false negative rate to be high (42 of 80 cases). This suggests that elevated MetO-Clusterin ratio levels do not always occur during the disease progression. The average background reading per sample accounted only for ~25% of the total measured value. The acquired data was confirmed by repetitive measurements (*n* = 3). Thus, it is clear from our data that relative to controls, higher MetO-Clusterin levels significantly correlates with human MCI and AD stages.

## 4. Discussion

A reliable, fast, and affordable blood test for AD diagnosis is an important objective to enable the effectiveness of available AD therapy at early stages of the disease. Several blood-based tests that showed good reliability and are based on the levels of the hallmark markers of AD (i.e., Aβ and phosphorylated tau and their various aggregate forms [7,8,9]) were developed. However, most of these tests lack either a marker that is not directly related to the AD hallmarks (these plasma AD markers not always correlate with the disease state) or are controversial in their interpretation without proper controls or the presence of APOE4 allele carriers. In the current study, we propose that MetO-Clusterin levels in human blood plasma may serve as an additional non-Aβ and phosphorylated tau corelative marker for the diagnosis of MCI and AD (Figure 1). The ability to monitor the levels of MetO-Clusterin levels in blood plasma through an Elisa kit facilitates a fast and cost-effective diagnosis. Based on other studies [10], just using the total clusterin levels in plasma as a marker for dementia or stroke is controversial, since age and other age-related factors may affect the correlation between the level of clusterin and its effect on the disease. The average age we used in our study for all groups is ~76 years, and we have not observed an age effect on the obtained data. In the future, we will gather samples from older individuals to provide further supportive evidence for the interpretation of our analysis. According to the ROC data analysis, it is expected that expanding the number of tested subjects in this MetO-Clusterin assay will provide better cut-off values (to all groups) and enhanced sensitivity and specificity values for the parameters used in the ROC analysis. If indeed so, the acquired data will improve the accuracy and specificity of the MetO-Clusterin assay and its impact as a clinical diagnostic tool for MCI or AD. The described Elisa assay to monitor relative levels of MetO-Clusterin in blood plasma is unique, since other assays monitor the hallmark markers of AD and MCI only (i.e., phosphorylated tau at position 217 (p-tau217) and the beta-amyloid ratio (Aβ42/40)), through the use of a combination of positron emission tomography (PET), Elisa, and mass-spec assays [9,10]. We have not developed an Elisa kit to monitor the ratio of MetO levels of a control blood plasma protein. This task involves the creation of a new Elisa kit that is specifically tailored to a suitable control protein and is complying with the sensitivity, specificity, and validity requirements of the assay. On one hand, if a control protein shows a similar pattern of methionine oxidation, like the data shown for MetO-Clusterin, it may foster new exploratory research about the connection of such protein to MCI and Alzheimer’s disease. On the other hand, if the chosen control MetO-protein will show no correlation with the disease, as shown with MetO-Clusterin, it may suggest that MetO-Clusterin levels are unique to their correlation pattern with the progression of MCI and Alzheimer’s disease. Overall, it is proposed that a combination of several AD-diagnostic blood plasma assays (including the one described in this study) will be used to more accurately diagnose MCI and AD in humans. It has yet to be determined whether an early rise in MetO-Clusterin levels in blood plasma can predict the occurrence of AD. We extensively collect blood samples, with the assistance of the KU AD Research Center, to enable us to monitor the levels of MetO-Clusterin in longitudinal studies, starting at the non-dementia stage through MCI and AD stages. A higher or change in the monitored levels of MetO-Clusterin in the longitudinal studies prior to the development of MCI and AD will greatly enhance the early diagnostics of AD that will be more successfully complemented by FDA-approved AD therapeutics. Furthermore, in future studies, we plan to acquire more samples that will both increase the number of participants in this study and expand their source/origin, thus allowing us to determine the effect of race/geographical origin on the data obtained.

## 5. Patents

J.M has a patent application related to this work.

## Figures and Tables

**Table 1:** Information about the subjects that participate in this study.

Diagnosis	Age (Years)	Sex, n	CDR-SOB Score	MoCA Score
**NL**	76.22 ± 4.5	Males, 21 Females, 23	0 ± 0.0	26 ± 2
**MCI**	76.11 ± 4.4	Males, 25 Females, 19	1.9 ± 1.0	21 ± 4
**AD**	76.61 ± 4.4	Males, 20 Females, 24	5.5 ± 2.5	16 ± 2

NL; non-dementia control; MCI: mild cognitive impairment; AD: Alzheimer’s disease; CDR-SOB: Clinical Dementia Rating. MoCA (Montreal Cognitive Assessment): a score that represents cognitive functions (visuospatial and executive functions, short-term and working memory, space orientation, and concentration ability).

## Data Availability

Further patients’ data is unavailable due to privacy or ethical restrictions.
